# First Report of Complete Sequence of a *bla*_NDM-13_-Harboring Plasmid from an *Escherichia coli* ST5138 Clinical Isolate

**DOI:** 10.3389/fcimb.2016.00130

**Published:** 2016-10-13

**Authors:** Jingnan Lv, Xiuqin Qi, Dan Zhang, Zhou Zheng, Yuehui Chen, Yinjuan Guo, Shanshan Wang, Liang Chen, Barry N. Kreiswirth, Yi-Wei Tang, Zengqiang Chen, Longhua Hu, Liangxing Wang, Fangyou Yu

**Affiliations:** ^1^Department of Laboratory Medicine, The First Affiliated Hospital of Wenzhou Medical UniversityWenzhou, China; ^2^Public Health Research Institute Tuberculosis Center, New Jersey Medical School, Rutgers UniversityNewark, NJ, USA; ^3^Department of Laboratory Medicine, Memorial Sloan Kettering Cancer CenterNew York, NY, USA; ^4^Department of Laboratory Medicine, The Second Affiliated Hospital of Nanchang UniversityNanchang, China; ^5^Department of Respiratory Medicine, The First Affiliated Hospital of Wenzhou Medical UniversityWenzhou, China

**Keywords:** *Escherichia coli*, *bla*_NDM-13_, plasmid

## Abstract

Since the first report of *bla*_NDM-1_, 16 *bla*_NDM_ variants have been identified among Gram-negative bacteria worldwide. Recently, a novel *bla*_NDM_ variant, *bla*_NDM-13_, was identified in the chromosome of an ST101 *Escherichia coli* isolate from Nepal. Here we first reported plasmid-mediated *bla*_NDM-13_ in a carbapenem-resistant *E. coli* ST5138 clinical isolate associated with hospital-acquired urinary tract infection from China. *bla*_NDM-13_ and *bla*_SHV-12_ coexisted on the a ~54 Kb self-transferable plasmid. Compared with NDM-1, NDM-13, NDM-3, and NDM-4 had two amino acid substitutions (D95N and M154L), one amino acid substitution (D95N) and one amino acid substitutions (M154L), respectively. Complete plasmid sequencing showed that *bla*_NDM-13_-harboring plasmid (pNDM13-DC33) was highly similar to the *bla*_NDM-1_-harboring IncX3 plasmid pNDM-HN380, a common *bla*_NDM_-harboring vector circulating in China. In accordance with the structure of pNDM-HN380, pNDM13-DC33 consists of a 33-kb backbone encoding plasmid replication (*repB*), stability partitioning, and transfer (*tra, trb*, and *pil*) functions, and a 21-kb antimicrobial resistance region with high GC content between *umuD* and *mpr* genes. In conclusion, the present study is the first report of a plasmid-encoded *bla*_NDM-13_ and the complete sequence of a *bla*_NDM-13_-harboring plasmid (pNDM13-DC33). *bla*_NDM-13_ maybe originate from *bla*_NDM-1_ located on a pNDM-HN380-like plasmid by sequential mutations.

## Introduction

*Enterobacteriaceae*, particularly *Escherichia coli* and *Klebsiella pneumioniae*, are common pathogens causing nosocomial infections. Carbapenems are the choice for the treatment of infections caused by multi-drug resistant *Enterobacteriaceae*, especially extended-spectrum β lactamase (ESBL)- and/or plasmid-mediated AmpC (pAmpC)-producing organisms (Tzouvelekis et al., 2012). The emergence of carbapenem-resistant *K. pneumonia* and *E. coli* producing carbapenemases (KPCs) and metallo-β-lactamases (MBLs) have become a major global health problem due to the limited number of effective antibiotic options to treat the infections caused by these multi-drug resistant *Enterobacteriaceae* (Tzouvelekis et al., 2012). In 2009, a novel MBL, named New Delhi metallo-β-lactamase-1 (NDM-1), was identified in a *K. pneumoniae* isolate from a Swedish patient who had returned from India with a urinary tract infection (Yong et al., 2009). Since then, NDM-1-producing Gram-negative isolates have emerged worldwide. NDM-1 was primarily identified in *Enterobacteriaceae*, especially in *E. coli* and *K. pneumoniae*, from the Indian subcontinent, Balkan states, the Arabian peninsula, and North Africa (Nordmann and Poirel, 2014). In China, NDM-1 was initially identified in 4 clonally unrelated *Acinetobacter baumannii* isolates in 2011(Chen et al., 2011). Subsequently, this clinically important enzyme has spread among many species of *Enterobacteriaceae* in China (Hu et al., 2013; Liu et al., 2013; Zhang et al., 2013).

Since the first report of NDM-1, 16 NDM variants have been identified among Gram-negative bacteria worldwide (http://www.ncbi.nlm.nih.gov/pathogens/submit_beta_lactamase/). Recently, a novel NDM variant, NDM-13, was reported in a multidrug-resistant *E. coli* clinical isolate in Nepal (Shrestha et al., 2015). The *bla*_NDM-13_ gene, interestingly, was found to locate within the chromosome of an *E. coli* ST101 isolate. The aim of the present study was to investigate whether *bla*_NDM-13_ was located on the plasmids of clinically isolated *Enterobacteriaceae*. We first found plasmid-mediated *bla*_NDM-13_ and completely sequenced a *bla*_NDM-13_-harboring plasmid for the first time from a carbapenem-resistant *E. coli* ST5138 clinical isolate associated with hospital-acquired urinary tract infection in China.

## Materials and methods

### Bacterial strain

From Mar, 2014 to Oct, 2014, a total of 87 carbapenem-resistant Enterobacteriaceae (CRE) isolates causing clinical infections isolated from various specimens of patients at the First Affiliated Hospital of Wenzhou Medical University in Wenzhou, east China, were investigated for carbapenemase genes. The isolates were identified as *E. coli* by an automated microbiology analyzer (bioMe'rieux, Marcy l'Etoile, France) in accordance with the manufacturer's instructions.

### Antimicrobial susceptibility testing

Gram-negative susceptibility (GNS) card on the Vitek system (bioMe'rieux, Marcy l'Etoile, France) was performed initially for antimicrobial susceptibility testing. Disk diffusion method was used for further confirmation and antimicrobial susceptibility results were interpreted according to the criteria recommended by Clinical and Laboratory Standards Institute (CLSI) (CLSI, 2014). The *E*-test method was used for the determination of minimum inhibitory concentrations (MICs) of imipenem and meropenem for the *E. coli* isolate and its transconjugant. *E. coli* ATCC 25922 was used as control strain for antimicrobial susceptibility testing.

### Detection of carbapenemases and extended-spectrum β-lactamases (ESBLs)

The modified Hodge test (MHT) was further performed on a Mueller-Hinton agar plate with ertapenem as substrate for the detection of carbapenemases as described previously (CLSI, 2014). MBLs were determined using a double-disc synergy test (Peleg et al., 2005). ESBLs were tested using the CLSI-recommended confirmatory double disk combination (CLSI, 2014).

### Detection of resistance genes

The carbapenemase genes responsible for carbapenem resistance, including *bla*_KPC_, *bla*_GES_, *bla*_SPM_, *bla*_IMP_, *bla*_VIM_, *bla*_SPM_, and *bla*_NDM_, were detected using PCR and DNA sequencing as described previously (Queenan and Bush, 2007; Nordmann et al., 2011). ESBLs genes were detected in accordance with the method described previously (Andrade et al., 2010). PCR products were analyzed by electrophoresis in 1% agarose gels and were sequenced on both strands.

### Transferability of plasmids with carbapenem resistance

In order to determine whether carbapenem resistance was transferable in *E.coli* DC33 strain, filter mating conjugation was performed using *E. coli* 600 as the recipient as previously described (Wang et al., 2004). Plasmid DNA of *E. coli* DC33 strain was extracted with the QIAGENPlasmid Midi kit (Hilden, Germany) according to the manufacturer's instructions. The plasmid extracts were transferred into *E. coli* DH5α by using chemical transformation and transformants were selected on Luria-Bertani agar plates containing imipenem (0.5 μg/ml).

### Multi-locus sequence typing (MLST)

Multi-Locus Sequence Typing (MLST) was performed on *E. coli* DC33 using amplification of internal fragments of the seven housekeeping genes including *adk, fumC, gyrB, icd, mdh, purA*, and *recA* of *E. coli* according to MLST website (http://mlst.warwick.ac.uk/mlst/dbs/Ecoli).

### Determination of *bla*_NDM-13_ location

The total bacterial DNA of *E. coli* DC33 was first prepared in agarose plugs, digested with S1 nuclease and further separated by pulsed-field gel electrophoresis (PFGE), as described previously (Chen et al., 2011). Then, the DNA bands were transferred horizontally to a nylon membrane (Millipore). A digoxigenin-labeled *bla*_NDM-13_ probe was used to hybridize with DNA bands and a nitro-blue tetrazolium/5-bromo-4-chloro-3′-indolylphosphate color detection kit (Roche Applied Sciences) was applied to detected hybridization signals.

### Sequencing a *bla*_NDM-13_-harboring plasmid from the transconjuguant of *E. coli* DC33 strain

In order to completely characterize the plasmid from the transconjugant of *E. coli* DC33 (designated as pNDM13-DC33), pNDM13-DC33 was isolated, purified, and sequenced using the Illumina MiSeq platform. The sequencing reads were *de novo* assembled, gaps between contigs were closed, open reading frames (ORFs) were predicted, and annotations were performed as described previously (Chen et al., 2013).

## Results and discussion

### Carbapenemases and ESBLs production and detection of resistance genes

Among 87 CRE isolates, 7 were positive for *bla*_NDM_. After sequencing, *E. coli* strain DC33 was found to harbor *bla*_NDM-13._ *E. coli* strain DC33 was isolated from a urine culture of a 64-year-old male hospitalized for prostatic hyperplasia in July, 2014. After hospitalized, the patient had the symptom of urinary tract infection. Subsequently, many white cells were found in urine sample under microscope. *E. coli* strain DC33 was isolated when the patient was hospitalized on day 8. *E. coli* DC33 was weakly positive for the MHT assay, but β-lactamase activity was inhibited by EDTA, indicating that *E. coli* DC33 produced a MBL. *E. coli* DC33 was also positive for CLSI-recommended confirmatory double disk combination test for detecting ESBLs. The results of detection of ESBL genes using PCR showed that *E. coli* DC33 was also positive for *bla*_SHV_ while was negative for other resistance genes tested. After DNA sequencing, *bla*_SHV_ was found to *bla*_SHV-12._

### Antimicrobial susceptibility testing

*Escherichia coli* DC33 exhibited resistance to all antimicrobials tested except tigecycline determined initially by Gram-negative susceptibility (GNS) card on the Vitek system (Table 1), including ampicillin, ampicillin/sulbactam, amikacin, aztreonam, cefotetan, ceftazidme, ceftriaxone, cefepime, ciprofloxacin, ertapenem, gentamicin, imipenem, levofloxacin, nitrofurantoin, piperacillin/tazobactam, tobramycin, and trimethoprim/sulfamethoxazole, The *E. coli* IOMTU558 carrying *bla*_NDM-13_ located on the chromosome from Nepal was highly resistant to all β lactams tested including ampicillin, ampicillin/sulbactam, cefepime, cefoselis, cefotaxime, cefoxitin, cefpirome, ceftazidime, ceftriaxone, cephradine, doripenem, imipenem, meropenem, and moxalactam (Shrestha et al., 2015). The *E. coli* IOMTU558 was also resistant to other antibiotics including ciprofloxacin, gentamicin, kanamycin, levofloxacin, and tobramycin, but susceptible to amikacin, colistin, fosfomycin, and minocycline (Shrestha et al., 2015). Tigecycline MICs for *E. coli* DC33 and *E. coli* IOMTU558 were 0.05 and 2 μg/ml (Shrestha et al., 2015). The antimicrobial susceptibility pattern of *E. coli* DC33 was further confirmed by disk diffusion method. The resistance of *E. coli* DC33 to imipenem and meropenem was further corroborated by *E*-test method.

**Table 1:** **MIC values of antimicrobials for ***E. coli*** DC33 carrying ***bla***_**NDM-13**_ and its transconjugant**.

**Antimicrobials**	**MIC values (μg/mL)**		
	**DC33**	**DC33-EC600**	**EC600**
Ampicillin	≥32	≥32	16
Ampicillin/Sulbactam	≥32	≥32	4
Piperacillin/Tazobactam	≥128	≥128	≤4
Cefotetan	≥64	≥64	≤4
Ceftazidime	≥64	≥64	≤1
Ceftriaxone	≥64	≥64	≤1
Cefepime	≥64	16	≤1
Aztreonam	≥64	≥64	≤1
Ertapenem	≥8	≥8	≤0.5
Imipenem	≥16	≥16	≤1
Amikacin	≥64	≤2	≤2
Gentamicin	≥16	≤1	≤1
Tobramycin	≥16	≤1	≤1
Ciprofloxacin	≥4	2	≤0.25
Levofloxacin	≥8	4	≤0.5
Nitrofurantoin	64	64	≤16
Trimethoprim/Sulfamethoxazole	≥320	≤20	≤20
Tigecycline	0.05	0.05	0.05

### MLST

MLST result showed *E. coli* DC33 belonged to ST5138, a single locus variant of ST617. Although ST5138 has been deposited in *E. coli* MLST database (http://mlst.warwick.ac.uk/mlst/dbs/Ecoli), no study about *E. coli* ST5138 isolate is published. In our previous study, coexistence of *bla*_NDM-1_ and *bla*_CMY-42_ was found among *E. coli* ST167 clinical isolates in our hospital (Zhang et al., 2013). As ST5138 was a single-locus variant of S167, we speculate that *E. coli* DC33 harboring *bla*_NDM-13_ is genetically related to *E. coli* ST 167 isolates carrying *bla*_NDM-1_ found in our previous study (Zhang et al., 2013). Recently, a Chinese study found an increasing prevalence of *E. coli* ST167 clinical isolates carrying both *bla*_NDM-1_ and *bla*_NDM-5_ on the conjugative IncX3 plasmid in various parts of China (Huang et al., 2016). Therefore, increasing emergence of *bla*_NDM_ variants among *E. coli* ST167 and ST167 variants clinical isolates should be of concern. Up to now, *bla*_NDM-13_ was only reported in Nepal (Shrestha et al., 2015). The present study is the second report of this novel *bla*_NDM_ variant.

### Location of *bla*_NDM-13_ gene and transferability of plasmids carrying *bla*_NDM-13_

**Figure 1:**
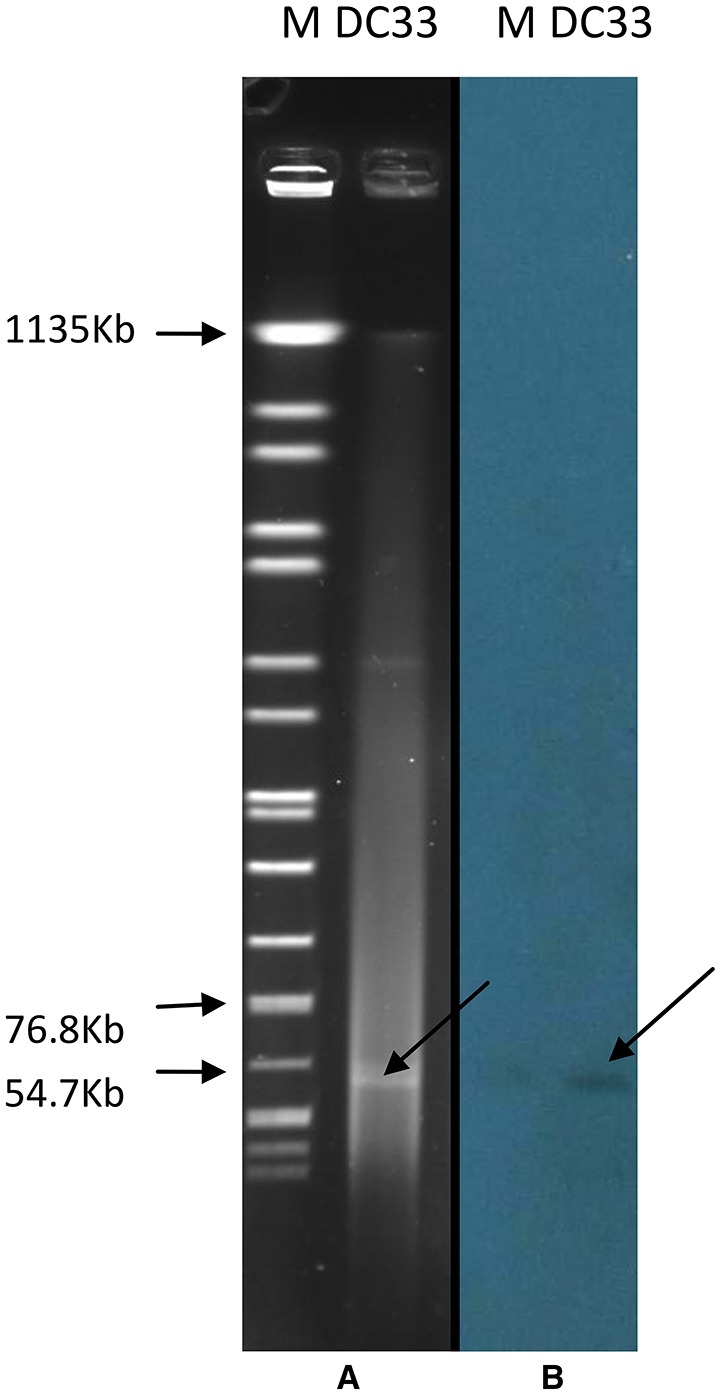
**S1-digested plasmid DNA of ***E. coli*** DC33 (A)**. Southern blot hybridization with *bla*_NDM-13_ of *E. coli* DC33 **(B)**. M, *Salmonella* serotype *Braenderup* strain H9812.

S1-PFGE result showed that a ~54-Kb plasmid was found in *E. coli* DC33 (Figure 1). Subsequently, *bla*_NDM-13_ gene was found to be located on this plasmid, not on chromosome, which was confirmed by Southern-blot (Figure 1). The *bla*_NDM-13_-harboring plasmid of *E. coli* DC33, designated as pNDM13-DC33, was successfully transferred into recipient *E. coli* 600 by filter mating conjugation. The antimicrobial resistance patterns of *E. coli* DC33 and its transconjugant were showed in Table 1. Shrestha et al found that *bla*_NDM-13_ was located within the chromosome (Shrestha et al., 2015). However, *bla*_NDM-13_ was first confirmed to be located on the plasmid in the present study.

### Complete sequence of pNDM13-DC33

**Figure 2:**
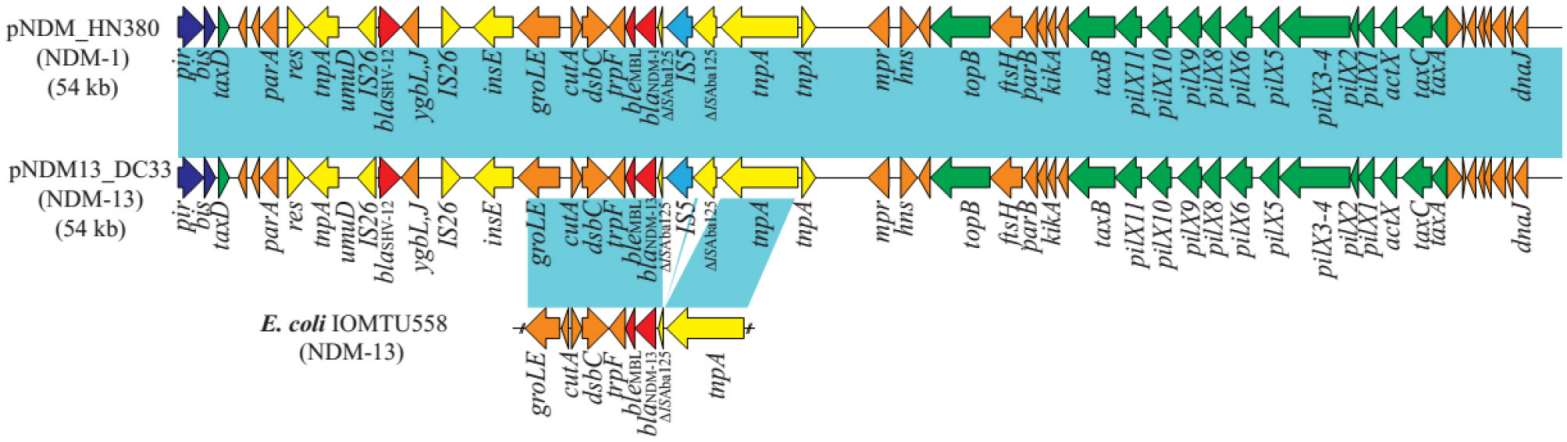
**Plasmid structures of pNDM_HN380 (JX104760), pNDM13_DC33(KX094555) and the ***bla***_**NDM-13**_ neighboring genetic environment in IOMTU558 (LC012596)**. Colored arrows represent open reading frames, with dark blue, yellow, green, red, light blue, and orange arrows representing replication genes, mobile elements, plasmid transfer genes, resistancegenes, IS*5*, and plasmid backbone genes, respectively. Blue shading denotes regions of shared homology among different plasmids.

Plasmid pNDM13-DC33 is 54.035-bp in length, with an average GC content of 49.03% (Figure 2). BLASTn analysis showed that pNDM13-DC33 is similar to pNDM-HN380, an IncX3-type plasmid carrying *bla*_NDM-1_ among *Enterobacteriaceae* isolates in China (Ho et al., 2012), with 100% query coverage and >99.9% nucleotide identity (with 8 single nucleotide polymorphisms, SNPs). In China, IncX3-type plasmids carrying *bla*_NDM_ variants have been widely found among *E. coli* clinical isolates with different clones including ST648, ST156, ST131, ST167, and ST3835 clones (Feng Y. et al., 2015; Huang et al., 2016; Wang et al., 2016; Yang et al., 2016). Notably, these similar plasmids have been identified in several hospitals from different geographic regions in China (Wang et al., 2014; Feng J. et al., 2015; Qu et al., 2015), suggesting that pNDM-HN380-like plasmids are common NDM vectors that likely contribute significantly to the dissemination of *bla*_NDM_ variants in China.

In accordance with the structure of pNDM-HN380, pNDM13-DC33 consists of a 33-kb backbone encoding plasmid replication (*repB*), stability partitioning, and transfer (*tra, trb*, and *pil*) functions, and a 21-kb antimicrobial resistance region with comparatively high GC content between *umuD* and *mpr* genes, suggesting that these two regions were likely acquired and genetically distinct. The resistance region of pNDM13-DC33, containing 16 ORFs sequentially organized as *IS26, bla*_SHV-12_, *ygbI, ygbJ, IS26, insE, groL, cutA1, dsbc*, Δ*trpF, ble*_MBL_, *bla*_NDM-13_, Δ*ISAba125, IS5*, Δ*ISAba125*, and Tn*3 tnpA*, was nearly identical to that of pNDM-HN380, but with the exception that they carry different *bla*_NDM_ variants (pNDM13-DC33 with *bla*_NDM-13_ and pNDM-HN380 with *bla*_NDM-1_) (Figure 2). Of note, compared with NDM-1, NDM-13, NDM-3, and NDM-4 had two amino acid substitutions (D95N and M154L), one amino acid substitution (D95N) and one amino acid substitutions (M154L), respectively (Table 2). Although NDM-13 (with two substitutions including the D95N and M154L relative to NDM-1) did not show increased hydrolytic activity against carbapenems, cephalosporins, and penicillins, it increased the affinity of NDM-13 for cefotaxime and affected the catalytic activity of the enzyme against cefotaxime (Shrestha et al., 2015). Our finding that *bla*_NDM-13_-harboring pNDM13-DC33 closely resembles *bla*_NDM-1_-harboring pNDM-HN380 provides evidence that novel *bla*_NDM_ variants emerge by sequential mutations of a pNDM-HN380-like plasmid carrying *bla*_NDM-1_.

**Table 2:** **Nucleotide and amino acid differences between NDM enzymes**.

NDM variants^*^	**Non-synonymous substitution**	**Amino acid substitution**
NDM-1	−	−
NDM-2	C82G	P28A
NDM-3	G283A	D95N
NDM-4	A460C	M154L
NDM-5	G262T, A460C	V88L, M154L
NDM-6	C698T	A233V
NDM-7	G388A, A460C	D130N, M154L
NDM-8	A389G, A460C	D130G, M154L
NDM-9	G454A	E152K
NDM-10	C94A, G107A, G205A, G220A, G598C	R32S, G36D, G69S, A74T, G200R
NDM-11	A460G	M154V
NDM-12	A460C, G665A	M154L, G222D
NDM-13	G283A, A460C	D95N, M154L
NDM-14	A389G	D130G
NDM-15	A460C, G698T	M154L, A233V
NDM-16	G262C, A460C, G698T	V88L, M154L, A233V

* *Nucleotide and amino acid positions (in comparison to NDM-1) of nonsynonymous substitutions were listed. Amino acid abbreviations follow the standard single letter code*.

The chromosomal organization of the *bla*_NDM-13_ gene initially found in the *E. coli* isolate IOMTU558 from Nepal was similar to that in pNDM13-DC33, except for a 260-bp deletion in *ISAba125* of 260 bp (353 to 94 bp upstream *bla*_NDM-13_ start codon). In contrast, the corresponding *ISAba125* on pNDM13-DC33 was in full-length (1087 bp), but was interrupted by the insertion of an IS*5* (at 265 bp upstream *bla*_NDM-13_ start codon) (Figure 1). A comparison of the chromosomal organization flanking *bla*_NDM-13_ in the *E. coli* isolate IOMTU558 (Shrestha et al., 2015) with plasmids harboring *bla*_NDM_ identified the a set of ordered genes, *tnpA*-IS*30-bla*_NDM-13_-*ble*MBL-*trpF-dsbC-cutA-groES-groL*, that were nearly identical in plasmid pPMK1 from Nepal, plasmidpKPX-1 from Taiwan, plasmid pNDM-MAR from Morocco, and in an *Enterobacter hormaechei* CCHB10892 plasmid from Brazil (Villa et al., 2012; Huang et al., 2013; Carvalho-Assef et al., 2014; Stoesser et al., 2014; Shrestha et al., 2015). This finding suggests that the chromosomal copy of *bla*_NDM-13_ may be the result of a rare integration event where a region of the plasmid recombined into the *E. coli* genome.

In conclusion, the present study is the first report of a plasmid-encoded *bla*_NDM-13_ and the complete sequence of a *bla*_NDM-13_-harboring plasmid (pNDM13-DC33). *bla*_NDM-13_ maybe originate from *bla*_NDM-1_ located on a pNDM-HN380-like plasmid by sequential mutations. The emergence of novel plasmid-mediated *bla*_NDM_ variants, originating through the mutations in *bla*_NDM_ from an epidemic plasmid, poses a concern that NDM variants with different β-lactamases hydrolytic activity will evolve.

### Nucleotide sequence accession number

The complete nucleotide sequences of plasmid pNDM13-DC33 has been deposited as GenBank accession no. KX094555.

## Ethical approval

The Ethics Committee of the first Affiliated Hospital of Wenzhou Medical University exempted this study from review because the present study focused on bacteria.

## Author contributions

JL, XQ, DZ, ZZ, YC, YG, and SW isolated bacteria and performed the laboratory measurements. FY and LW made substantial contributions to conception and design. LC, YT, and BK revised the manuscript critically for important intellectual content. LC and JL participated in experimental design and data analysis. FY drafted the manuscript. All authors read and approved the final manuscript.

## Funding

This work was supported in part by National Institutes of Health (NIH) Grant R01AI090155 (to BK) and R21AI117338 (to LC). The content is solely the responsibility of the authors and does not necessarily represent the official views of the NIH.

### Conflict of interest statement

The authors declare that the research was conducted in the absence of any commercial or financial relationships that could be construed as a potential conflict of interest.
